# First report of transmission of canine leishmaniosis through bite wounds from a naturally infected dog in Germany

**DOI:** 10.1186/s13071-016-1551-0

**Published:** 2016-05-10

**Authors:** Torsten J Naucke, Silke Amelung, Susanne Lorentz

**Affiliations:** Parasitus Ex e.V., Vollbergstraße 37, 53859 Niederkassel, Germany; Department of Zoology, Division of Parasitology, University of Hohenheim, 70599 Stuttgart, Germany; Laboklin GmbH & Co. KG, Steubenstraße 4, 97688 Bad Kissingen, Germany; Kleintierpraxis Amelung, In der Schart 1, 52222 Stolberg, Germany

**Keywords:** Canine leishmaniosis, Dog-to-dog transmission, Bite wounds, Germany

## Abstract

**Background:**

Canine leishmaniosis (CanL) is an important zoonosis caused by *Leishmania *(*L.*) *infantum*. Transmission of *L. infantum* to dogs (and humans) is mainly through the bite of infected sandflies, but the parasite can also be transmitted vertically, venereally and through blood transfusions of infected donors. Additionally, the direct dog-to-dog transmission through bites or wounds is suspected.

**Results:**

In December 2015, a female eight-year-old Jack-Russell-Terrier was tested positive for CanL in Germany (ELISA 74, IFAT 1:4.000). The dog had never been in an endemic area, had never received a blood transfusion and had never been used for breeding.

Another female Jack-Russell-Terrier (born 2009 in Spain) was kept in the same household between 2011 and 2012. That dog was imported to Germany in 2011 and was tested positive for leishmaniosis in 2012. The Spanish-born dog had received several bite wounds, i.a. in the neck, during fights with the German-born Terrier.

**Conclusion:**

This may be the first report of transmission of *L. infantum* through bite wounds from a naturally infected dog in Germany.

## Background

Canine leishmaniosis (CanL) is an important zoonotic disease caused by the blood and tissue dwelling protozoan parasite *Leishmania *(*L.*) *infantum*. The domestic dog is considered the primary reservoir host for zoonotic leishmaniosis in endemic regions [1]. The main route of transmission of the parasite to dogs (and humans) is via the bite of the female phlebotomine sandfly. The vector ingests the parasite while blood-feeding, and then transmits the infective stages during a following blood meal.

Other than the insect route, CanL can be transmitted vertically and venereally [2–4] and through transfused blood products from infected donors [5, 6]. A suspected mode of transmission is the direct dog-to-dog transmission of the parasite by wounds or dog bites [7–10].

For the future this might be especially relevant for non-endemic countries without known vectors where the number of infected dogs is increasing owing to journeys to endemic areas or the import of infected animals.

CanL is a systemic disease that may potentially involve any organ, tissue or body fluid and is often manifested by nonspecific clinical signs [11]. The clinical course varies from an asymptomatic infection to a life-threatening generalized disease. Skin lesions are the most frequent manifestations. However, dogs can be presented with other clinical signs unrelated to cutaneous lesions [12]. Other common clinical presentations are renal, ocular and articular lesions. In the majority of cases lymphadenomegaly, lethargy, emaciation and muscular atrophy is observed. Chronic proteinuric nephritis that may progress to end-stage kidney disease is the main cause of mortility due to CanL [13].

Common laboratory abnormalities include hyperproteinemia observed with hypergammaglobulinemia, hypoalbuminemia and anaemia [14, 15].

The diagnosis of CanL can be made by the detection of specific serum antibodies using quantitative serological techniques, such as the immunofluorescence antibody test (IFAT) and enzyme-linked immunosorbent assay (ELISA) [16]. A high level of antibodies together with clinical signs and clinicopathological abnormalities compatible with leishmaniosis confirms the diagnosis of CanL [17].

In this report we describe the first possible dog-to-dog transmission of CanL through bite wounds in Germany.

## Findings

### Dog A

In December 2015, an 8-year-old female Jack-Russel-Terrier (Dog A; born October 2007) was presented in a veterinary practice in Germany. The owner had observed that the dog had become lethargic and inactive.

The dog was born in Germany (Mayen, Rhineland-Palatinate) and is kept in Stolberg (Rhineland). It has travelled to Slovakia (Pezinok; June 2013), Austria (Lamprechtshausen, a municipality in the Austrian state of Salzburg; May 2013), northern France (Dinard on the French Atlantic coast; July 2014) and several times to Sweden during summer (Värmland).

Although the dog has never been in an endemic area, the veterinarian decided to test for viral and bacterial infections including leishmaniosis, not least because another *Leishmania*-infected dog had been kept in the same household a few years earlier.

Serologic tests, conducted in December 2015, included an enzyme-linked immunosorbent assay (ELISA, cut-off value > 5 antibody units; ELISA based on soluble promastigote antigen in combination with immunoglobuline G(γ)-specific conjugate [18]) and an indirect fluorescent antibody technique (IFAT, cut-off value > 1:50, MegaScreen FLUOLEISH®, MegaCor, Austria). Because IFAT sensitivity and specificity are near 100 % in symptomatic dogs, the test is considered by the World Organization for Animal Health (OIE-Office International des Epizooties) as a reference serologic method [19].

The serum sample of the bitch was tested positive for antibodies against *Leishmania infantum* (ELISA 74, IFAT 1:4.000).

**Fig. 1:**
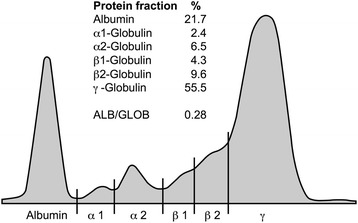
Serum electrophoretic patterns in Dog A. The dog was tested positive for antibodies against *Leishmania* (IFAT, ELISA). Serum protein electrophoresis revealed a characteristic hypergammaglobulinemia

To confirm the diagnosis of CanL, serum protein electrophoresis was carried out. The laboratory studies revealed a hyperproteinemia (93.9 g/l, reference interval 54–75 g/l), a hypergammaglobulinemia (55.5 %, reference interval 8–18 %), a hypoalbuminemia (21.7 %, reference interval 47–59 %), and a decreased albumin/globulin-ratio (0.28, reference interval 0.59–1.11), characteristic features of CanL (Fig. 1).

Complete blood count revealed anaemia with decreased RBC count and decreased hematocrit and hemoglobin levels (RBC 5.30 10^6^/μl, reference interval 5.50–8.50 10^6^/μl; HCT 34.6 %, reference level 44.0–57.0 %; HGB 11.2 g/dl, reference interval 15.0–20.0 g/dl).

The dog was treated with Allopurinol (15 mg/kg body weight per day) and Domperidon (5 mg/day) with a good clinical response.

### Dog B

From 2011 to 2012, another female Jack-Russel-Terrier (Dog B) was kept in the same household as Dog A. Dog B was born 2009 in Spain and was imported to Germany in the beginning of 2011. Dog B had several fights with Dog A, which resulted in wounds in Dog B as reported by the owner and the primary care veterinarian. The dog showed no other signs of disease until January 2012.

Shortly after a fight with Dog A in January 2012, Dog B was referred to the university clinic by the primary care veterinarian because of vomiting, diarrhoea, oedema in the legs and head, apathy, and anorexia. Uraemia (blood urea nitrogen 64.1 mg/dl, reference level 9–29 mg/dl), highly elevated serum creatinine level (serum creatinine 3.2 mg/dl, normal range <1.4 mg/dl) and hyperphosphatemia (inorganic phosphate 6.3 mmol/l, reference level 0.9–1.7 mmol/l) were diagnosed. A urine test strip revealed high amounts of blood and protein. Complete blood count revealed anaemia with (slightly) decreased hematocrit and hemoglobin levels (RBC 5.53 10^6^/μl, reference interval 6–9 10^6^/μl; HCT 39 %, reference level 38–55 %; HGB 12.4 g/dl, reference interval 15.0–19.0 g/dl). Serum biochemical analysis highlighted a hypoproteinemia (4.8 g/dl, reference interval 5.5–7.3 g/dl) and a hypoalbuminemia (2.03 g/dl, reference interval 3.1–4.6 g/dl).

A polymerase chain reaction (PCR) for *Leishmania* ssp. in blood was negative, while serology for *Leishmania* spp. antibodies was positive (30 units; reference values: <7 units negative, 7–12 units borderline, >12 units positive) (commercial ELISA kit, afosa GmbH; standard PCR was performed by a modified PCR protocol [20]). Cytological findings in the left and right *Lymphonodus praescapularis* showed a small number of macrophages infiltrated with *Leishmania* amastigotes and free *Leishmania* bodies.

Despite intensive treatment at the university clinic, the general condition of the dog worsened considerably within 24 h. In consultation with the veterinarian the dog owner decided to euthanize the dog.

## Discussion

The described canine leishmaniasis case corroborates the possibility of direct dog-to-dog transmission of CanL in a non-endemic country. But several hypotheses can be considered to explain the mode of transmission.

The distribution of CanL is greatly related to the distribution of appropriate vectors. In Europe, CanL is known to be endemic in countries surrounding the Mediterranean Basin, namely Albania, Croatia, southern France (the clinical prevalence in northern France is close to 0 % [21]), Greece, Cyprus, Italy, Malta, Portugal, Spain and Turkey [21, 22].

When CanL is diagnosed in dogs in non-endemic areas, it is usually in individuals that have travelled or resided in endemic areas. According to conservative estimates, there are 20,000 infected dogs currently in Germany [23].

All seropositive *L. infantum*-infected dogs, whether they express clinical disease or not, are potential sources of infection for vectors and may transmit the parasite [21]. Since studies have provided evidence for the natural occurrence of sandflies also in non-endemic European areas, the possibility of the transmission of the parasite by the bite of the natural vector must be taken into consideration [21, 23–25].

While *P. ariasi* and *P. perniciosus* are proven sandfly vectors of *L. infantum*, *P. mascittii* has not yet been confirmed as a vector, but its competence is suspected [23].

*Phlebotomus *(*P.*) *perniciosus* was detected near the German city Kaiserslautern (Rhineland-Palatinate). In addition, various specimens of *P. mascittii* were caught in different locations in Baden-Wurttemberg and one specimen near Cochem on the River Mosel [23, 24].

In France, the species *P. perniciosus, P. mascittii* and *P. ariasi* have been identified outside the endemic Mediterranean regions [21]. Recently, the occurrence of *P. mascittii* was documented in Austria, or more specifically in Styra, Burgenland and Lower Austria [25]. Phlebotomine sandflies have not been found, so far, in the Nordic countries and Slovakia [21].

The fact that dog A has never been in an endemic country/area does not fully exclude a possible transmission of CanL via sandflies; however, it is only with the smallest of probabilities that an infected sandfly transmitted the infection.

Cases of CanL in non-endemic areas might also occur as a result of non-sandfly transmission. Reported modes of non-vectorial transmission include vertical and venereal transmission as well as infections through transfused blood products from infected blood donors [2–6]. Dog A had never been used for breeding and had never received any blood transfusions.

Since a blood sample of Dog A’s mother is not available for the authors, a transplacental transmission cannot be fully excluded. In naturally infected dogs, subclinical infection may persist for months or years. Detailed knowledge about the incubation period of vertically infected dogs is limited due to a lack of representative data. But the authors hypothesize that the clinical signs would have occurred earlier in Dog A (eight years old) after a transplacental infection.

Additionally, direct dog-to-dog transmission through bites or wounds has been suspected to be a possible reason for the spread of *L. infantum* in foxhounds in the USA [7, 8] and recently for non-vector-borne transmission of CanL in dogs in Finland and New Caledonia Island [9, 10].

Also in this case report, the possibility of transmission of the parasite via dog bites with blood to blood contact cannot be excluded even if the dogs involved show no clinical signs of disease. *Leishmania* spp*.* can be isolated from intact skin of asymptomatic dogs [26]. And even though infectiousness of dogs increases with clinical severity [27], asymptomatic dogs or dogs after successful therapy (dogs never reach parasitological cure) probably act as reservoirs in the transmission of *Leishmania* parasites [28, 29].

Taken together, the data presented here has epidemiological significance since *Leishmania*-infected dogs may represent a risk of infection for domestic dogs even in the absence of natural vectors. Further research is needed to detect the exact mechanisms and rates of direct dog-to-dog transmission.
